# Microstructure and Mechanical Properties of Ti-25Nb-4Ta-8Sn Alloy Prepared by Spark Plasma Sintering

**DOI:** 10.3390/ma15062158

**Published:** 2022-03-15

**Authors:** Ilona Voňavková, Filip Průša, Jiří Kubásek, Alena Michalcová, Dalibor Vojtěch

**Affiliations:** Department of Metals and Corrosion Engineering, University of Chemistry and Technology Prague, Technická 5, 166 28 Prague, Czech Republic; filip.prusa@vscht.cz (F.P.); jiri.kubasek@vscht.cz (J.K.); alena.michalcova@vscht.cz (A.M.); dalibor.vojtech@vscht.cz (D.V.)

**Keywords:** titanium alloy, β-titanium, Ti-25Nb-4Ta-8Sn, spark plasma sintering, low modulus

## Abstract

As the commercially most-used Ti-6Al-4V alloy has a different modulus of elasticity compared to the modulus of elasticity of bone and contains allergenic elements, β-Ti alloy could be a suitable substitution in orthopedics. The spark plasma sintering (SPS) method is feasible for the preparation of materials, with very low porosity and fine-grained structure, leading to higher mechanical properties. In this study, we prepared quaternary Ti-25Nb-4Ta-8Sn alloy using the spark plasma sintering method. The material was also heat-treated in order to homogenize the structure and compare the microstructure and properties in as-sintered and annealed states. The SPS sample had a modulus of elasticity of about 63 ± 1 GPa, which, after annealing, increased to the value of 73 ± 1 GPa. The tensile yield strength (TYS) of the SPS sample was 730 ± 52 MPa, ultimate tensile strength (UTS) 764 ± 10 MPa, and ductility 22 ± 9%. Annealed samples reached higher values of TYS and UTS (831 ± 60 MPa and 954 ± 48 MPa), but the ductility decreased to the value of 3 ± 1%. The obtained results are discussed considering the observed microstructure of the alloy.

## 1. Introduction

Recently, interest in the research of β-Ti alloys has significantly grown. Commercially used cp-Ti (commercially pure titanium) and Ti-6Al-4V alloys are not ideal materials. Relatively high modulus of elasticity and presence of Al and V are the main limitations in the long-term implantation of cp-Ti and Ti-6Al-4V alloys [1,2,3,4]. The modulus of elasticity of Ti-6Al-4V alloy is about 110 GPa, and the modulus of elasticity of human bone is approximately 10–45 GPa [3]. This mismatch between the modulus of elasticity leads to the so-called stress-shielding effect. The bone is insufficiently loaded and begins to resorb. This process is painful for the patient and can result in the loss of the implant. If these problems begin to occur, the implant must be removed from the patient. Further surgery is stressful for the patient, and it increases the cost of the health care system. Another problem is the presence of Al and V in the alloy. During the life of the implant, the abrasion of the material occurs, and the microparticles are released into the human body. These particles can cause allergic reactions and can accumulate in the body tissues. To save the expenses of the health care system, and to reduce patient suffering, Al- and V-free β-titanium alloys have been developed. Nontoxic elements that stabilize the β modification of titanium (or do not affect modifications) were chosen as alloying elements, such as Nb, Mo, Ta, Zr, and Sn [1,3]. Many binary (Ti-Nb [1,5,6,7,8], Ti-Zr [9,10,11]), ternary (Ti-Nb-Ta [12], Ti-Nb-Zr [13,14,15], Ti-Zr-Mo [9], Ti-Nb-Sn [16,17]), and quaternary (Ti-Nb-Zr-Mo [18], Ti-Nb-Ta-Sn [19], Ti-Nb-Zr-Sn [20,21], Ti-Nb-Ta-Zr [3,4,22,23]) systems were studied. These β-titanium alloys exhibit a lower modulus of elasticity, higher strength, better corrosion resistance, and superior biocompatibility, compared with cp-Ti or Ti-6Al-4V.

As mentioned above, the Ti-Nb-Ta-Zr system (TNTZ) has been studied by many researchers [3,4,22,23]. These alloys show a relatively low modulus of elasticity (50–65 GPa) and contain only biocompatible elements. Niobium is an effective β-stabilizing element that suppresses the formation of α-phase. The strength of the alloy is maintained by the formation of a solid solution of Nb in Ti. The addition of niobium helps to reduce the modulus of elasticity and improves the corrosion resistance of the alloy by forming niobium oxides in the passive layer [3]. Tantalum also improves corrosion resistance due to the forming of tantalum oxides, which increases the protective effect of the passive layer. Minor addition of zirconium can contribute to improvement in mechanical properties, but the content of Zr should not exceed 6 at. % [15]. Another alloying element, Sn, plays a significant role in reducing the modulus of elasticity of the alloy by suppressing the formation of metastable ω-phase. Moreover, Sn contributes to the strengthening of the alloy by a solid-solution strengthening mechanism [24]. However, higher contents of Sn may increase the modulus of elasticity or disrupt the corrosion resistance [25,26].

Moraes et al. [25] reported that the addition of Sn significantly affects the mechanical properties of Ti-30Nb-xSn (x = 0, 2, 4, 6, 8, and 10 wt.%) alloy. The alloy was arc-melted and cast in a copper mold. The Vickers hardness and modulus of elasticity values showed decreasing trends up to 6 wt.% of Sn, and then the values increased again. Moreover, the compression yield strength and ultimate compression strength decreased with increasing Sn content, but alloys with 10 wt.% Sn reached the highest values. The maximum value of ductility was achieved with 6 wt.% of Sn, and the consequent increase in content resulted in a reduction in ductility. The martensitic α”-phase gradually disappeared with increasing Sn content, and the Ti-30Nb-8Sn and Ti-30Nb-10Sn alloys contained only β-phase. The highest corrosion resistance was achieved by the addition of 6 wt.% Sn.

Recently, more advanced manufacturing techniques of preparation of these alloys, such as 3D printing, spark plasma sintering (SPS), etc., have been studied. All these methods enable relatively fast production from initial powders than conventional methods (casting, forging, machining, etc.). The SPS process is used to compact the initial powders at lower temperatures and produces a material with high density, fine-grained structure, and high mechanical properties. The principle of this method is a combination of pressing and heating caused by the simultaneous passage of electric current pulses through the material (up to 20 kA). Due to this phenomenon, the initial powder, which is placed in conductive graphite die, is rapidly heated (up to 1000 K/min). Owing to the high sintering speed and pressure, materials with almost theoretical density and fine-grained structure can be prepared. Through this process, significantly better mechanical properties of the material can be achieved [27,28]. In this study, we used the spark plasma sintering technique to prepare a quaternary Ti-25Nb-4Ta-8Sn alloy. Compared with the more commonly investigated TNTZ alloys, we replaced the more expensive zirconium with cheaper tin, in consideration of possible future use and economic aspects. To date, few studies have been performed on this alloy.

Bartáková et al. [19] studied the Ti-Nb system with Zr, Ta, and Sn additions prepared by arc melting process with subsequent thermomechanical processing (hot forging, cold swaging, and solution treatment). They reported that the Ti-Nb-Ta-Sn alloy has the lowest modulus of elasticity (about 45 GPa). The tensile strength of this alloy reached 820 MPa. The microstructure of this alloy consisted of a β-Ti phase structure, with a small amount of α-Ti phase along the grain boundaries.

Thus far, to the best of our knowledge, there are several studies dealing with β-Ti alloys prepared by the SPS method [3,4,15,29,30,31]. None of these studies deals with the Ti-Nb-Ta-Sn alloy. Kong et al. [31] studied Ti-13Nb-13Zr alloy prepared by mechanical alloying and spark plasma sintering. The sintered alloy was composed mainly of β-phase with uniformly dispersed α- and α”-phases. The alloy achieved compression strength of about 1 790 ± 10 MPa, with plastic strain 16.5% and Vickers hardness of 356.7 ± 3.6 HV 10, at sintering temperature 900 °C.

## 2. Materials and Methods

**Figure 1:**
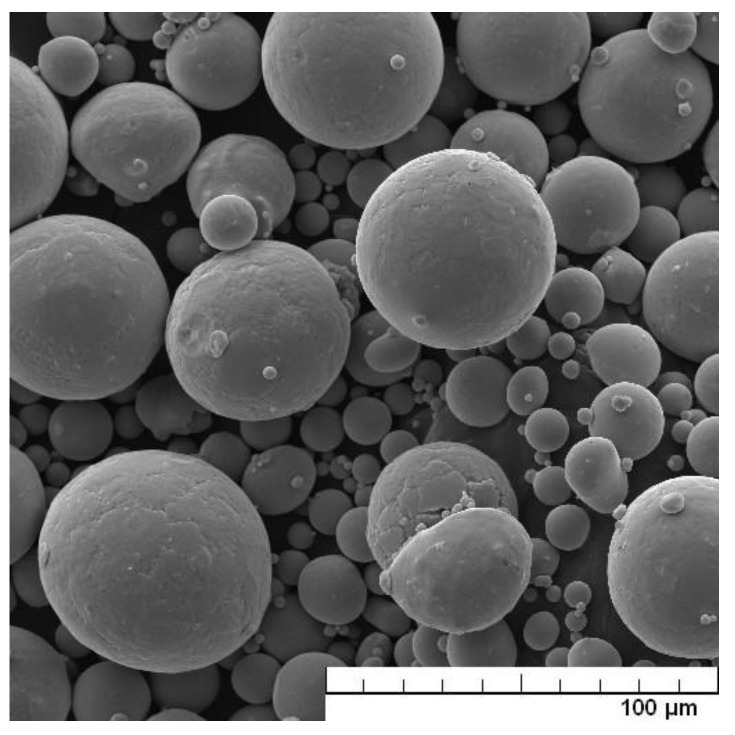
Morphology of the initial powder of Ti-25Nb-4Ta-8Sn alloy prepared by gas atomization (SEM).

**Figure 2:**
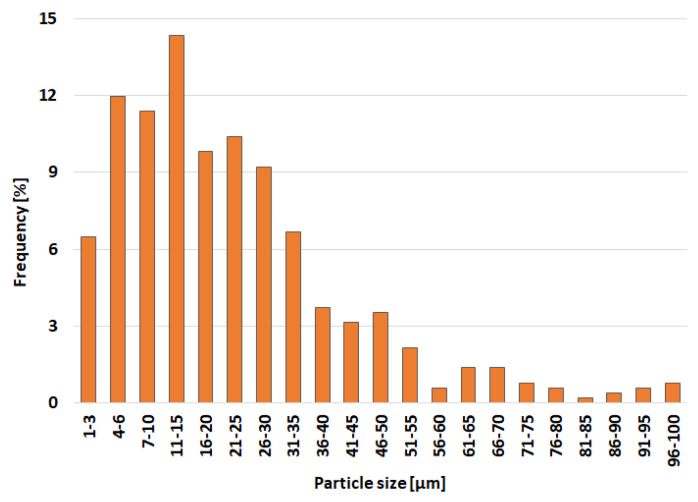
Distribution of particle size of initial Ti-25Nb-4Ta-8Sn powder.

The pure metals were mixed with a composition of 25 wt.% Nb, 4 wt.% Ta, 8 wt.% Sn, and the rest was Ti. Metals were melted, and the prepared alloy was subsequently gas-atomized. The morphology of the atomized powder particles is shown in Figure 1. The particle size of the atomized powder was determined from SEM images by image analysis. At least 500 particles were measured, and the distribution of the particle size of the initial powder is shown in Figure 2.

The atomized powder of Ti-25Nb-4Ta-8Sn alloy was processed using the spark plasma sintering (SPS) method with an FCT Systeme HP-D 10 machine (FCT Systeme GmbH, Rauenstein, Germany). The material was heated up to 1000 °C, with a heating rate of 200 °C/min. The holding time at this temperature was 10 min, and the applied pressure was 80 MPa. The final product was a tablet-shaped sample with a diameter of 20 mm and a height of 9 mm. The chemical composition of the alloy was confirmed by X-ray fluorescence (XRF). The sample was divided by cutting into smaller parts, and these were used for microstructure studies and compression tests. Other tablets were prepared for tensile tests with the same processing conditions. The material was further heat-treated in order to optimize microstructure and properties. Annealing was performed in a resistance furnace at 600 °C for 1 h in an ambient atmosphere. A low temperature was chosen to prevent excessive oxidation, and a short time to prevent grain coarsening. Designation of the samples used in the following text and processing parameters are summarized in Table 1.

For microstructure studies, the sample was embedded in a metallographic resin, ground on SiC abrasive papers P80–P2500, and polished on diamond paste D2 (Urdiamant, Šumperk, Czech Republic). Final polishing was performed with a 5:1 mixture of Eposil F (colloidal silica, Metalco Testing, Roztoky u Prahy, Czech Republic) and hydrogen peroxide. To highlight the microstructure, the sample was etched in Kroll’s reagent consisting of 5 mL HNO3, 10 mL HF, and 85 mL distilled water. The microstructure was observed by an optical microscope (Olympus PME3, Tokyo, Japan) and by scanning electron microscopes (SEM, Tescan Vega3 LMU and Tescan Lyra3, Brno, Czech Republic) equipped with an energy-dispersive spectrometer (EDS, Oxford Instruments Aztec software, High Wycombe, UK). Porosity was evaluated from optical microscope images by image analysis using ImageJ software. The phase composition was determined by X-ray diffraction (PANalytical X’Pert PRO, Cu Kα source λ = 0.1540 nm, Almelo, Holland). The sample for TEM observations was prepared by grinding to thin foil on SiC papers and then processed by a precision ion-polishing system (Gatan PIPS, Berwyn, PA, USA). The microstructure of the so-prepared sample was subsequently observed using a transmission electron microscope (TEM, Jeol JEM 2200 FS, Tokyo, Japan; EDS, Oxford Instruments Aztec 3.0, High Wycombe, UK).

**Figure 3:**
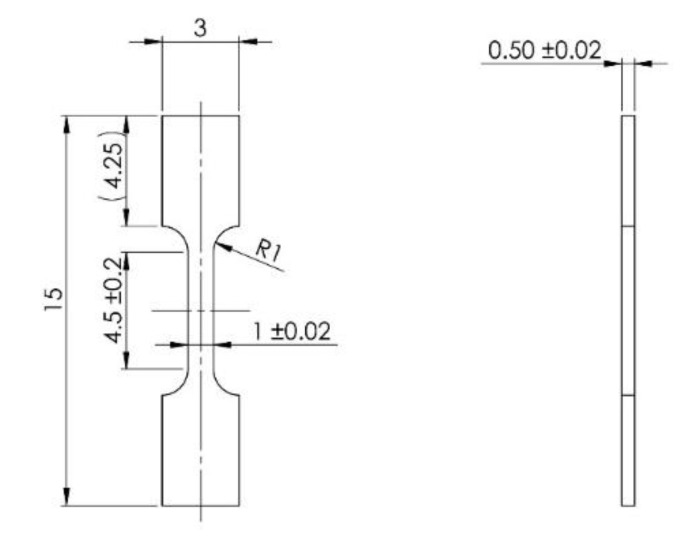
Dimensions of microsamples for tensile test (in mm).

The microhardness of the material was measured using a microhardness tester FUTURE TECH FM-700 (FUTURE-TECH CORP., Kawasaki, Japan), with a load of 1 kg. Compressive and tensile tests were performed to determine the mechanical properties. For the implementation of mechanical compression tests, 3 samples in the shape of a square-based block with dimensions of a = 3.3 mm and h = 4.7 mm were prepared. Compression tests were carried out using the universal testing machine LabTest 5.250SP1-VM (LaborTech, s.r.o., Opava, Czech Republic) at the temperature of 25 °C. The strain rate for the compression test was 0.001 s^−1^. For tensile tests, bone-shaped microsamples, with dimensions shown in Figure 3, were prepared from the sintered tablet. The tests were performed on a Tira tester with a constant speed of 0.0675 mm/min at a temperature of 25 °C.

## 3. Results

### 3.1. Microstructure

**Figure 4:**
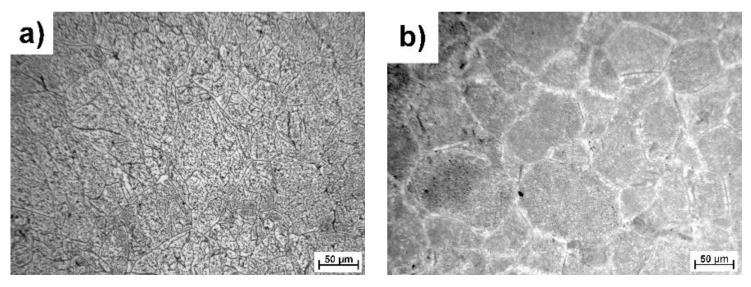
Microstructure of Ti-25Nb-4Ta-8Sn alloy prepared by SPS (**a**) and after heat treatment (**b**) (optical microscope).

**Figure 5:**
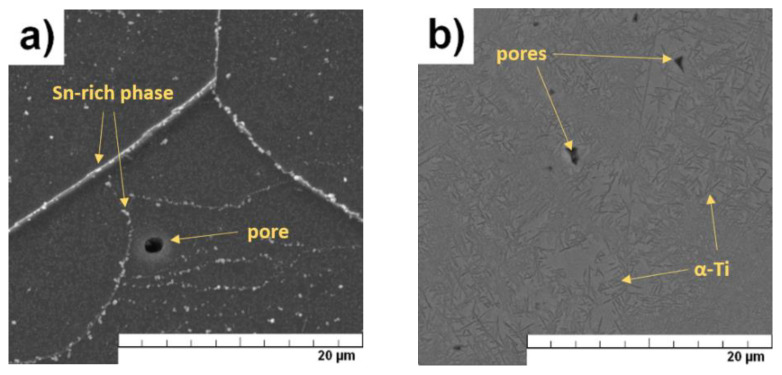
Microstructure of Ti-25Nb-4Ta-8Sn alloy prepared by SPS (**a**) and after heat treatment (**b**) (SEM).

The Ti-25Nb-4Ta-8Sn alloy was successfully prepared by the SPS method. XRF confirmed that the chemical composition of the alloy was 24.4 ± 0.1 wt.% Nb, 3.8 ± 0.1 wt.% Ta, 7.6 ± 0.2 wt.% Sn, and the rest was Ti. Optical microscope images are shown in Figure 4, and more detailed SEM images are shown in Figure 5. The microstructure of the SPS sample consisted of relatively large β-Ti grains and intermetallic phases that precipitated on the grain boundaries and inside the grains during the SPS process (Figure 5a). The porosity of the SPS sample was 1.8 ± 0.4%. From Figure 4b, it can be seen that annealing led to grain coarsening.

**Figure 6:**
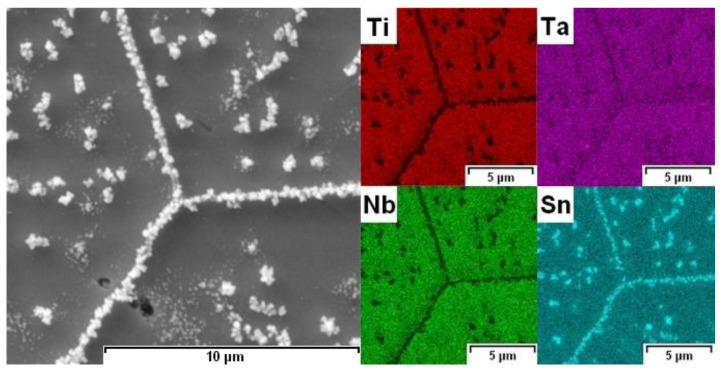
Elemental mapping images of SPS sample (SEM–EDS).

EDS analysis (elemental mapping images) revealed that the precipitated phases in SPS material were rich in Sn (Figure 6). Due to the size of the phases (<1 µm) and the signal-generating area of the SEM–EDS (2–3 µm), more detailed point analysis was influenced by signals from the surroundings of the phases. Bartáková et al. [19] studied the Ti-25Nb-4Ta-8Sn alloy prepared by arc melting process, followed by hot forging (700–1100 °C) and solution treatment (850 °C/0.5 h/furnace cooled). In the microstructure, they observed only β-Ti grains with a very small fraction of α-Ti phase, especially along the grain boundaries. No Sn-rich phase was detected in their study.

**Figure 7:**
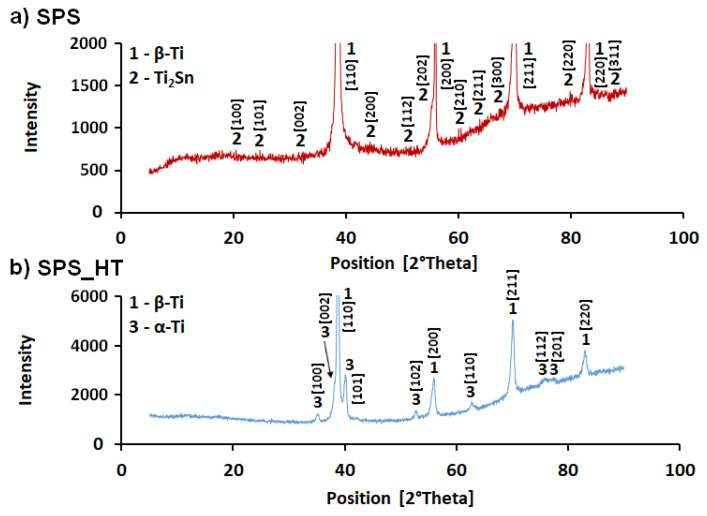
Diffraction patterns of Ti-25Nb-4Ta-8Sn alloy: SPS sample (**a**) and heat-treated sample (**b**).

X-ray diffraction analysis of the SPS sample proved the presence of β-Ti phase (BCC, a = b = c = 0.329 nm) in the alloy, and the phase containing Sn was identified as Ti_2_Sn phase with hexagonal crystal lattice (a = b = 0.474 nm and c = 0.563 nm), as shown in Figure 7. Since the β-Ti phase was dominant in the structure, the peaks of the Ti_2_Sn phase showed a very low intensity. The Ti_2_Sn phase was revealed by a more detailed examination of the measured data in the X’Pert HighScore program. The presence of this phase was also indicated by EDS analysis, which showed the content of 71.7 ± 2.8 at. % Ti and 28.3 ± 2.8 at. % Sn in the analyzed particles. Slightly higher content of Ti was caused by the surrounding Ti matrix and small dimensions of Ti_2_Sn particles.

**Figure 8:**
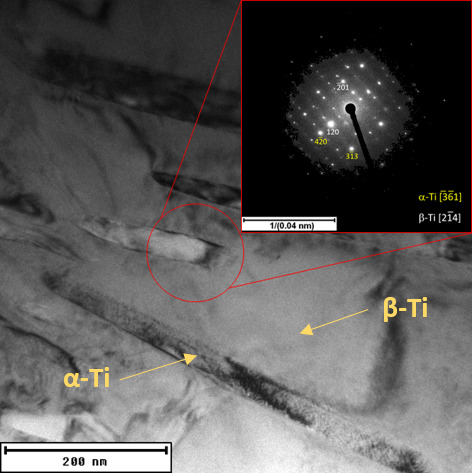
Detailed microstructure of SPS_HT sample with needle-like α-Ti phase in β-Ti matrix (TEM).

**Figure 9:**
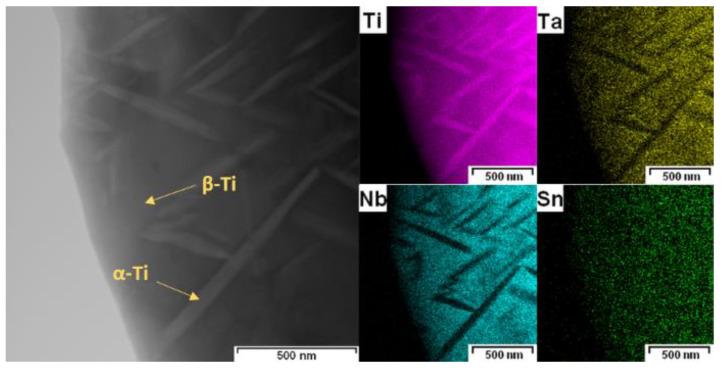
Elemental mapping images of SPS_HT sample (TEM–EDS).

During annealing, the Sn-rich phase dissolved, and the β-Ti phase (BCC) partially transformed into the more stable α-Ti phase (HCP, a = b = 0.296 nm, c = 0.476 nm), as shown in Figure 5 and Figure 7. SPS_HT sample contained approximately 78% of β-Ti phase and 22% of α-Ti phase. The precipitated α-Ti phase is clearly visible in the TEM image (Figure 8). Electron diffraction analysis proved that the needle-like phase was α-Ti, with a hexagonal close-packed crystal lattice. Furthermore, the α-Ti phase contained less niobium than the β-Ti phase (9.0 ± 0.7 wt.%, 25.9 ± 0.7 wt.%, respectively), which was confirmed by EDS point analysis and elemental mapping images (Figure 9). Niobium, as well as tantalum, is a β-stabilizer, and thus it dissolved more in β-Ti than in α-Ti.

### 3.2. Mechanical Properties

Vickers microhardness of the SPS sample reached 293 ± 5 HV 1. After annealing, the microhardness only slightly increased to 305 ± 2 HV 1 (Table 2). Due to the precipitation of the fine α-Ti phase during annealing (Figure 9) and lower porosity, the alloy was slightly strengthened despite the grain coarsening. Furthermore, solid solution strengthening may play a role due to the dissolution of the Sn-rich phase.

**Figure 10:**
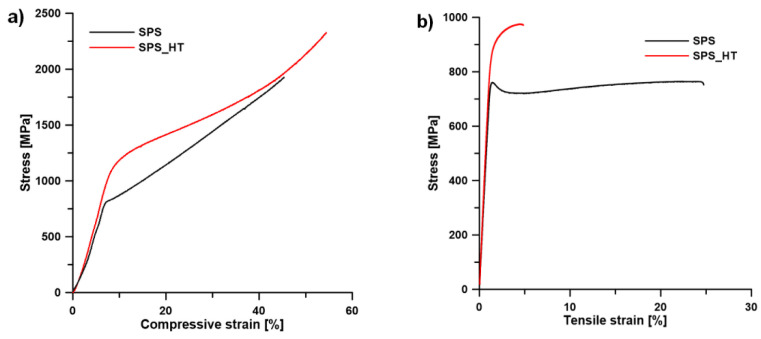
Compressive stress–strain curves (**a**) and tensile stress–strain curves (**b**).

A similar trend was observed after compression and tensile tests (Figure 10 and Table 2). The yield strength increased after annealing by more than 100 MPa, both in compression and in tension. Both samples (SPS and SPS_HT) behaved very plastically, and no fracture was observed during the compression tests (Figure 10a). For this reason, only the compressive yield strength (CYS) could be determined. In tension, the SPS material was plastic with ductility (A) of more than 20%. After heat treatment (SPS_HT) the material became more brittle (Figure 10b), and the ductility was only 3 ± 1%. The reason is in the precipitation of very fine α-phase particles (Figure 8 and Figure 9). The modulus of elasticity (E) of the SPS sample was 63 ± 1 GPa. After annealing, the modulus of elasticity increased to a value of 73 ± 1 GPa because of α-Ti phase precipitation.

The same alloy was studied in [19], and the tensile test was performed. They achieved the best combination of low modulus of elasticity (43 GPa), good ductility (about 18%), and relatively high strength (820 MPa). Our SPS sample had finer grains and no α-Ti phase in the structure. In the study of Bartáková et al. [19], the hot-forged + solution-treated sample exhibited higher strength than the SPS sample in this study because of the presence of the α-phase, which causes the strengthening of the alloy. However, after annealing, we achieved a higher tensile strength of 954 MPa. The SPS Ti-25Nb-4Ta-8Sn alloy had a higher modulus of elasticity (~63 GPa) than that measured by other authors [19], which can be caused by the presence of the Ti_2_Sn phase. This phase was not observed in Ti-Nb-Ta-Sn alloys by other authors, so its effect on the properties of β-Ti alloys is not known.

In [25], Moraes et al. studied the effect of Sn addition to as-cast Ti-30Nb alloy. The addition of Sn up to 6 wt.% resulted in the lowest Vickers hardness, lowest compressive strength, and maximum value of ductility. With increasing Sn content, ductility significantly decreased. The Ti-30Nb-8Sn alloy showed Vickers hardness of about 230 HV, which is lower than that of the SPS Ti-25Nb-4Ta-8Sn sample of this study. They also conducted a nanoindentation test to reveal the modulus of elasticity of the alloy. The modulus of elasticity of the Ti-30Nb-8Sn alloy was much higher (about 80 GPa) than the modulus of elasticity of the SPS sample (63 GPa). Table 3 provides a comparison of the mechanical properties of selected alloys prepared by different processes.

## 4. Conclusions

The Ti-25Nb-4Ta-8Sn alloy was successfully prepared using the spark plasma sintering (SPS) method. The material was studied in sintered (SPS) and annealed states (SPS_HT). Both samples exhibited very low porosity (1.8 ± 0.4%, 0.6 ± 0.1%, respectively). The sintered sample consisted of a solid solution of alloying elements in the β-Ti and Ti_2_Sn phases. After annealing at 600 °C/1 h, the Ti_2_Sn phase dissolved, and the α-Ti phase precipitated. This resulted in strengthening and hardening of the alloy, despite the grain coarsening. Additionally, the modulus of elasticity increased from 63 to 73 GPa value, but it is still a significantly lower value than that of commercially used Ti-6Al-4V alloy. Unfortunately, the ductility decreased from 22% to 3% due to annealing. The SPS Ti-25Nb-4Ta-8Sn alloy could be a suitable substitution for Ti-6Al-4V alloy, but further studies need to be conducted to achieve the best combination of strength and plasticity.

## Figures and Tables

**Table 1:** Designation of samples and processing parameters.

Sample Designation	Processing Parameters
SPS	SPS (1000 °C/10 min)
SPS_HT	SPS (1000 °C/10 min) + annealing (600 °C/1 h)

**Table 2:** Specific values of mechanical properties in compression and tension.

Sample	Compression Test				Tensile Test			Vickers Hardness
	CYS (MPa)	UCS (MPa)	ε (%)	TYS (MPa)	UTS (MPa)	E (GPa)	A (%)	HV 1
SPS	818 ± 7	−	−	730 ± 52	764 ± 10	63 ± 1	22 ± 9	293 ± 5
SPS_HT	1033 ± 7	−	−	831 ± 60	954 ± 48	73 ± 1	3 ± 1	305 ± 2

ε = relative deformation, A = ductility, E = modulus of elasticity.

**Table 3:** Comparison of mechanical properties of selected alloys in tension.

Alloy	Processing	Microstructure	TYS (MPa)	UTS (MPa)	A (%)	E (GPa)	Ref.
Ti-25Nb-4Ta-8Sn	cast → hot-forged → heat-treated → water-quenched→ cold-swaged	β	~750	820	~18	~43	[19]
Ti-25Nb-4Ta-8Sn-0.4O		β	~1150	~1150	8	68	[19]
Ti-32Nb-2Sn	cast -→ hot-rolled → solution-treated → water-quenched	β	665 ± 25	780 ± 5	32 ± 2	60 ± 2	[16]
	cast → hot-rolled → solution-treated → water-quenched → aged (500 °C/6 h)	α + β	960 ± 5	1070 ± 15	8 ± 1	82 ± 2	[16]
	cast → hot-rolled → solution-treatment → water-quenched → aged (600 °C/6 h)	α + β	560 ± 30	685 ± 20	7 ± 3	−	[16]
Ti-25Nb-11Sn	cast → hot-forged → hot-rolled → cold-swaged → heat-treated (400 °C/2 h)	β	1300	1330	~8	~86	[17]

## Data Availability

Not applicable.
